# Global policymakers and catastrophic risk

**DOI:** 10.1007/s11077-021-09444-0

**Published:** 2021-12-02

**Authors:** Christopher Nathan, Keith Hyams

**Affiliations:** Politics and International Studies, University of Warwick, England, UK

**Keywords:** Emerging technology, Biotechnology, Artificial intelligence, Global catastrophic risk

## Abstract

There is a rapidly developing literature on risks that threaten the whole of humanity, or a large part of it. Discussion is increasingly turning to how such risks can be governed. This paper arises from a study of those involved the governance of risks from emerging technologies, examining the perceptions of global catastrophic risk within the relevant global policymaking community. Those who took part were either civil servants working for the UK government, U.S. Congress, the United Nations, and the European Commission, or cognate members of civil society groups and the private sector. Analysis of interviews identified four major themes: Scepticism; Realism; Influence; and Governance outside of Government. These themes provide evidence for the value of conceptualising the governance of global catastrophic risk as a unified challenge. Furthermore, they highlight the range of agents involved in governance of emerging technology and give reason to value reforms carried out sub-nationally.

## Introduction

The growing literature on global catastrophic risks (herein, ‘GCR’) examines hazards that are ruinous, or terminal, to the whole of humanity. Despite its recent progress in understanding issues around the governance of emerging technology, this literature lacks empirical studies of the outlook of those who may influence it. In particular, there is a dearth of empirical studies, whether qualitative or quantitative, of those who might, in the very near future, be involved in enacting the reforms that are being proposed. Our interest in this paper is with the governance of risks arising from emerging technologies, particularly in the perceptions and attitudes of relevant policymakers, and in how far obstacles to effective governance this area are based in fundamental structural problems with political institutions.^1^ We show how interviews with such participants can shed critical new light on the problem of how to govern GCR effectively. Our aim is to improve understanding of current perceptions of GCR governance among those actually or potentially involved in such governance. We seek to develop a snapshot of the current state of play that goes beyond what is described in policy documents, and sheds light on how relevant actors perceive what is going on in practice. We explore not only on the extent to which those involved in GCR governance perceive that such governance is or is not successful, but also identify the sources of problems and potential remedies. This is of value in determining what is feasible, in getting a greater understanding of where unidentified issues exist, in seeing how proposals can best be executed, and in understanding what aspects of governance call for more research.

The study of global catastrophic risks is maturing as an independent interdisciplinary field. The case has been made for paying concerted attention to these risks (Bostrom, 2013), including but not limited to those who argue that there is very strong moral reason to pay attention to the effects of our actions upon future generations (Greaves & MacAskill, 2019; Ord, 2020).^2^ The literature has examined specific risks to the survival of humanity or a large part of it, such as threats from pandemics, biotechnology, super volcanoes, artificial intelligence (AI), and nuclear war. It has also made efforts to compare these risks and to seek out possible new ones (for recent overviews and assessments see Ord, 2020; Beard & Torres, 2020). The field has also become more conceptually sophisticated. It has examined more closely what relevantly unites cases of ‘global catastrophic risk’. It has drawn attention to the way that the concept is intertwined with ideas of resilience (Avin et al., 2018). And it has noted how apparently separate risks may combine into catastrophic risks (Liu et al., 2018; Kuhleman, 2018).

Attention is also turning to specific policy proposals and forms of governance that may better address these risks. Posner (2004) provides a systematic set of reasons for why there is insufficient movement in addressing catastrophic risk, setting out the culture and psychology of confronting high impact, low probability risks, as well as the economic context, including the decentralisation of the global political system. Nick Bostrom (2019) argues that unknown threats from new technologies provide a strong reason in favour of a world government and unprecedented surveillance of individual citizens, and that less draconian measures would be unlikely to mitigate the threat sufficiently (see also Manheim, 2020, but compare Caplan, 2008). Bruce Tonn (2018) also considers what level of risk would warrant ubiquitous controls upon society, or the placing of a state on a war footing. Less drastically, it has been proposed that existing elements of international law can be harnessed so that states can collectively undertake a commitment to govern risks to humanity from emerging technologies (Wilson, 2013). There remain interesting opportunities for further conceptual development about policy, including drawing on studies in organisational theory examining the way that social structures create risks, and how we are led to attribute failures to individuals rather than the structure (Gephart, 2004; Perrow, 2011; Pidgeon & O’Leary, 2000).

Some scholars take an economic perspective, conceptualising the risk from emerging technology as an externality—a cost of doing research that is not borne by any actor in a market. The cost, in this case, is in part an information hazard: the chance that knowledge resulting from research might, once discovered, itself be a danger to humanity (Bostrom, 2011). Examples are the theory behind nuclear fission in the 1930s, or, now, the method for synthesising a highly dangerous and infectious pathogen. If research has these external costs, we might therefore seek ways to intervene in the market so that these costs are placed upon individuals, perhaps by placing taxes or other limits upon certain kinds of research (Farquhar et al., 2017; Posner, 2008; Taylor, 2008).

There is also a set of proposals involving institutional nudges. For example, the various ‘Commissioners for Future Generations’ involve mandates for lawmakers to consider the effects of legislation upon future generations (see Jones et al., 2018 for an overview). The central motivation behind such proposals is that a part of the explanation for our failure so far to adequately address threats from emerging technologies lies in psychology. A series of biases means that we fail to make effective assessments of risks that have a low probability but a high impact (Yudkowsky, 2008; Wiener, 2016). For example, given our tendency to assess probabilities in a frequentist way, counting up the instances in which an outcome has occurred in the past and comparing it to some reference class, we will tend to fail to give due to possible outcomes in which humanity does not exist.^3^ It may be that institutions can be arranged better to counter these biases and encourage more long-term, risk-aware thinking on the part of those with power (see also Caney, 2016; Baum, 2016; Marchant & Allenby, 2017). We might also note broader studies of the public in general, including Schubert et al (2019), which suggests the people do not consider the end of humankind to be uniquely bad, in comparison to non-existential catastrophe, and Tonn (2009), suggesting that people do not think that existential risks will eventuate in the next 500 years.

Of course, these overarching proposals sit alongside proposals and discussions concerning specific aspects of emerging technology. For example, some argue for particular reforms to the way that DNA synthesis orders are met (Esvelt, 2018); some urge community oversight of gene editing (Kofler et al. 2018); some urge legal personhood for artificial intelligence (Turner, 2018); some seek to facilitate and harness the research community-led regulation of Artificial General Intelligence projects, particularly through the recent rush of AI ethics guidelines (Jobin et al., 2019; Bellfield, 2020).^4^ Such proposals exist against a complex and incomplete set of governance instruments at domestic and international levels. Recent studies have mapped these structures and identified gaps and issues requiring attention—notably, Kemp and Rhodes (2020; see also Wilson, 2013).

There are relevant studies of the relation between science and policy in broader or adjacent contexts. Some describe how a ‘technoscientific viewpoint’ is evidenced by interviews with science policymakers. This perspective involves subjecting and apparently reducing ideological or value choices to complex technical decisions, thereby abdicating responsibility for difficult choices, or obscuring the grounds for making them (Macnaghten & Chilvers, 2014; Smallman, 2020; Wynne, 1993, 2001). More broadly, Ulrich Beck, beginning with and following from *The Risk Society* (1992; Beck, 2009), argues that rapid technological advances have brought with them the manifest possibility of humanity doing great damage, and that this in turn places us in a qualitatively new era, a second, ‘reflexive’ modernity.

Although not threats from *emerging* technology, there are relevant studies in the areas of nuclear weapons and climate change mitigation. In nuclear policy, there are a number of reports of interviews with high-ranking officials (Kurokawa, 2019; Yoshida, 2018) and perhaps because of its highly centralised nature, discussions on that topic often draw upon interviews with such individuals (Khan, 2014; Lentner, 1976; Sagan, 1996), although there is also a movement towards quantitative studies in this field (Gartzke & Kroenig, 2016; Geller, 2017). Of particular relevance to the present paper is Frank Sauer’s (2015) work on ‘atomic anxiety’, which deploys a qualitative study of publicly available recordings of US presidential decision-making, and shows how the taboo on the use of nuclear weapons can be detrimental to deterrence.

A number of studies in climate policy use semi-structured interviews in order to understand what inhibits senior policymakers from implementing more effective climate change mitigation policies. Simonet and Leseur (2019) author a study focused on French local authorities, identifying five main barriers to effective climate adaptation policy, referring to resources, skill levels of officials, awareness within local authorities, broader prioritisation of climate change, and regulatory requirements. More broadly, Rickards et al (2014) and Hambira and Saarinen (2015) give analyses of what constrains policymakers from taking decisive action on climate change mitigation, even in the face of their acceptance of the threat. Rickards et al. focuses on the social position of senior decision-makers, arguing that ‘even very well-intentioned individuals can struggle to exert considerable change while maintaining their positions [as senior decision makers]’ (ibid., p. 765). Stedman (2004) is an online survey (*n* = 851) of the perceptions of the policy community of risk from climate change, finding a wide diversity of attitudes. Bauer et al. (2012) draw on research and interviews in order analyse the types of policy considered by policymakers for climate change mitigation, noticing a leaning towards ‘soft, voluntary forms of coordination or steering’ (ibid., p. 300), against a background in which it is challenging to create a globally coherent approach.

Overall, the literature on governance of global catastrophic risk—and governance of risk more broadly—points to factors bearing on the success or failure of governance arrangements as occurring at three different levels. Rickards et al (2014), set out the following terminology to identify each of these levels:

- the micro-level, referring to individual and interpersonal factors
- the meso-level, referring to organisational and institutional factors
- the macro-level, referring to societal, cultural, and economic factors.^5^

The framework provided by this categorisation provides the analytical background for our study. Although the micro–meso–macro-distinction is more commonly used to refer to levels of analysis, rather than factors affecting a system, recent work in public administration urges that the relations between the different levels should be theorised (e.g., Roberts, 2020), and that we can understand where within the theorised system a given phenomenon sits. Clearly, the individual policymaker action, the institution, and the social and political context are interdependent. For example, a specific initiative by a policymaker is enacted within general cultural trends, and some initiatives can have impact upon those trends. Interview questions were selected in order to probe both obstacles and opportunities at each level of governance. Our aim was both to explore the views of policymakers in order to confirm issues hypothesised in the GCR literature as bearing on governance, and also to look for new concerns or opportunities not previously identified.

What we offer here is distinct because it has both a focus on the topic of global hazards from *emerging* technology, and is evidenced with analysis of interviews with those in power. Of particular interest is the question, to what extent are deficiencies in existing GCR governance arrangements linked to more fundamental structural problems with global and national political governance—for example inbuilt incentives for short-termism? How we answer this question will be of central importance for thinking about potential remedies for problems with existing GCR governance arrangements (or lack thereof). To the extent that problems do not appear to be linked to more fundamental issues with political structures, it may be possible to achieve effective GCR governance by fostering new institutions or approaches to governance that do not require more deep seated change. On the other hand, to the extent that concerns about GCR governance arise from deeper difficulties with global and national political governance, we may be forced to confront these broader issues in order to make progress.

## Method

Participants were sought purposively, with a diversity across levels of seniority, institution-type, and technology focus, covering (a) civil servants working for the UK government, U.S. Congress, the United Nations, and the European Commission, and (b) members of civil society groups and the private sector with an interest in the areas covered. We narrow the field to those with policy roles relating to two specific areas of emerging technology, namely, AI and biotechnology. This enables a balance of, on one hand, the value of examining policy across respondents within a particular field, with, on the other hand, the value of identifying what issues resonate across fields, such as the need for long term policymaking. Both areas show potential for remarkable progress in the near future, given breakthroughs such as the discovery of gene drives and the recent achievements of neural networks. We focus on these two areas as a way of making the study more feasible and facilitating analysis across correspondents, while also, given their differences, allowing reflection on governance culture of risks from emerging technology in general. Our approach is, as Robinson (2014) puts it, to ‘delineate a sample universe that is coherent with [researchers’] aims and questions and with the research resources they have at their disposal.’ The concept of a ‘global policymaker’ covers a range of different types of actor. We include not just civil servants serving national governments and international governing bodies, but broader civil society, because we give the term ‘governance’ a wide definition, in accordance with the trend towards seeing government as a process of problem-solving interaction between public and private actors (Kooiman, 2003; Pierre & Peters, 2020). That is, we take the stance that understanding the way that the field is governed is furthered by an understanding of the broader policy context in which government agents operate.

Our approach to selecting interviewees was to begin with trusted contacts and direct contact with relevant organisations, followed by the use of snowballing, an approach that most directly facilitates the involvement of senior and directly relevant participants. Through this method we selected 16 respondents who represent the spectrum of stakeholder types involved in defining and implementing policy in this area, and who would be expected together to provide a rich account of the topic.^6^ All participants’ roles covered the governance of artificial intelligence or biotechnology. As it is an open question how far GCR should be governed in such terms, not all participants work directly on catastrophic threats. Given the exploratory nature of this study, the aim was to allow for in-depth exploration with a select number of often elite interviewees rather than a large scale survey of the field. The process by which we obtained participants had the strong advantage that it allowed us, through building on trusted connections, to include some very senior policymakers. Although probability-based sampling was not possible in this context, we sought to include a balance of participants across levels of seniority, technology type, and organisation type. We are confident that the data we have is robust enough to generate useful conclusions given its richness. Ethical approval was provided by [redacted for anonymous review].

Interviews were recorded, professionally transcribed, and assigned pseudonyms. Interviews were carried out from late 2019 through to 2020. Each lasted between thirty and eighty minutes. Semi-structured interviews were deployed, with a topic guide setting open questions on the participants’ professional work; their views of the dangers of technology within a 50–100 year timespan; the efficacy of current governance with respect to those dangers; the obstacles to good governance; and the role of democratic and stakeholder-engaged processes. Each of these questions within the topic guide was supplemented with possible follow-up questions, designed to obtain more detail where appropriate.

Several participants sent in advance of the interview a relevant report that they had been involved in writing; in these cases, the report would form a background to the interview and would shape further questions within the protocol. There is a possible tension arising in studies of those working as experts in niche areas. On one hand, greater background knowledge on the part of the interviewer provides an opportunity for a more probing exchange and thereby a richer data set. On the other hand, by becoming invested in each subject, the interviewer may increasingly take particular stances. Indeed, in order to avoid the latter effect, studies sometimes explicitly involve researchers postponing the work of carrying out a literature review until after the interviews are completed (Anderson & Clarke, 2019). In this study, in contrast, prior technical knowledge was highly valuable, although in all cases it remained natural to frame the participant as the expert during interviews.

The interviews were analysed within the software package NVivo 12 with what Braun and Clarke (2006, 2019) call ‘reflexive thematic analysis’. This is an especially appropriate method because it can be used to find patterns in diverse languages and experiences. All transcripts were carefully read in order to obtain researcher familiarity. The first coding was then carried out with a view to being open to what ideas may be at work across the data. Some codes were based on technology types or topic areas, while most were analytic ideas about the subject. Coding was carried out reflexively, meaning that the codebook was developed and adjusted in tandem with the coding, in accordance with the goal of respecting the idea that ‘new meanings are always (theoretically) possible’ (Braun and Clarke 2021: 210). After the initial coding, codes were combined or placed within hierarchies, in search of themes. Themes go beyond codes; they capture ‘something important about the data in relation to the research question,’ and furthermore, represent a ‘patterned response or meaning within the data set’ (Braun & Clarke, 2006: 82). In this case, they capture something about our question, ‘What are the perceptions of governance of global catastrophic risk within the relevant global policymaking community?’ The process was inductive: rather than testing pre-established hypotheses, the themes from the data are brought out in the process of analysis.^7^ Theme development occurred in stages, with possible themes being noted as the analysis progressed, and codes being gradually rearranged where they overlap or form hierarchies in order to demonstrate the themes. For example, in the analysis here, the code ‘scepticism’ subsumed the codes ‘optimism’ and ‘reactiveness’, and the latter subsumed ‘disaster as motivator’. These codes together suggested the theme ‘scepticism,’ with its own shape and exceptions. Similarly, the code ‘policymaker norms’ subsumed ‘anti-idealism,’ ‘policy conservatism,’ and ‘language’ which itself subsumed ‘GCR brand’ and ‘policy concepts.’ These together suggested the ‘influence’ theme. The themes were then each interrogated for the story that they tell, and the report writing phase extracted quotations that illustrate each of the themes.

Four themes are presented here: ‘Scepticism’, ‘Realism’, ‘Influence’, and ‘Governance outside of Government’. These themes are generated by the research but are also guided by the questions that we wished to answer in the light of gaps in background literature, as described above. First, the Scepticism theme arises from an emergent picture of how effective policymakers consider existing GCR governance arrangements to be. Second, the theme that is ultimately labelled ‘Realism’ arises from the connections between problems with GCR governance and more fundamental issues with national and international political governance. The third theme, Influence, arises from a perceptions of the scope of policymakers to bring about change in order to achieve more effective governance of GCR. Finally, Governance-outside-of-Government brings out the potential for new modes of GCR governance to be developed which, as we will see, appeared to several interviewees to be the most promising route forward.

## Findings

### Theme one: scepticism

There is widespread scepticism about the efficacy of current governance of global catastrophic risks. In short, the view is taken that existing policies are inadequate. As one UN policy lead on health security puts it in an interview in early 2020 regarding pandemics ‘if a crisis hits tomorrow, it’s too late’ (12).^8^ An author of AI policy, pressed on what would occur were artificial intelligence rapidly to develop powerful and potentially malign capabilities, states, ‘if you had a…big crisis now that involved AI…at an international kind of scale, I’m not sure…what the response would be’ (04). One thing that stands out is how this attitude of scepticism is put forward across different governance and technology types and areas.

Furthermore, several take the view that existing governance is highly reactive. That is, in order for more efficacious systems to be put in to place, it is necessary for disasters of some magnitude to eventuate. An interviewee in a policy role working for a major AI company stated that the company would be ‘likely to remain in reactive mode’ regarding the issue of public perceptions of political influence upon the company (01). A senior expert with government advisory roles (07) presciently states in an interview in late 2019:

when you think about Homeland Security in the United States for example, it was really after September 11^th^ that we changed the way that we thought about the threats. And I fear that that’s how the global community is going to approach it with pandemic preparedness. Something’s going to happen, and then it’s going to be obvious why we should taken it more seriously. But of course, after that, it’s too late.

The scepticism, then, extends beyond assessments of the existing structures, and often in to assessments of the modes of change in which the existing structures can engage.

Scepticism might be up front and put forward as part of a case for improvement, or implicit, especially coming from those whose work is not currently directly focused on governance of GCR. It was not the central job of every participant in the study to focus directly on GCRs, and it may be said that those whose roles did not involve this took a less optimistic view of the ability to govern this area effectively.

As one might expect from the policy community, the scepticism narrative tends to involve not just diagnoses but also prognoses and treatments. One influential expert on emerging biotechnology policy (02) states:

I’m definitely not optimistic…about our governments being able to create more prospective efficient governing systems in the time that they need to be. And I have some hope in scientists doing better, but I’m actually not all that optimistic for them either just because they’re still working in systems that really are in direct conflict with doing better for society.

The tendency is to set out some of the systematic problems facing those seeking to put in place effective governance mechanisms. The scepticism theme is thereby accompanied with some version of the following two themes. Before continuing to these, we note a subtheme of ‘Scepticism’, which we may call the *Problem Solving Mindset*. Consider the following exchange, which took place in the early days of the Covid-19 crisis. The interviewee, who works on UK research policy, had just spent some time answering a question about the threats from future technology.

Interviewer: Am I right thinking that wouldn’t have been in the answer that you might have given, say, 12 months ago?

(19): No, …[Covid-19] is something that clearly has stress tested the UK, the whole system … there will be more focus on what…could happen, … [T]he learning that I have had is that having a small root that’s dynamic… that isn’t hampered by too much red tape, is important, but is linked directly into central government…making sure that whatever the product or process that’s been developed is absolutely required and not being afraid to stop when the data shows that things have changed…

Here, we see an instance of a pattern that occurs in several interviews: the problem-solving mindset turns attention in the discussion quickly from possible threats to achievable actions, to areas that can be solved, especially by expanding on existing governance practices. In this case, the interviewee turns the question to what policy lessons there are to take from the response to Covid-19. This is a departure from scepticism about existing governance practices; the implicit lesson is that wholesale assessments of governance practices are not useful exercises.

### Theme two: realism

Policymakers tend to be acutely aware of the status of GCR within global politics and emphasise the complexity involved in addressing it. In response to a question about the efficacy of governance of emerging technology, one interviewee working in private sector government relations role expressed the view that, on one hand, effective governance would have to be created by those who hold a great deal of power, while, on the other hand, such changes may be unlikely, given what we are referring to here as macro-issues in current geopolitics (01). Similarly, our health security policy expert (12) states:

there’s also the realpolitik because…you can have as many agreements as you want, but if you’ve got the UK and the United States producing something, politically, their first obligation is clearly their own citizens because they’ll be skewered if they had to do anything else, so it raises some very difficult questions that are still not solved.

Another interviewee with experience of high-level work at the United Nations laments the way that multi-stakeholder governance within the UN context tends to be undermined by state bodies, referring to the ‘tenaciousness of member states as the provider of mandates and the provider of resources. It always surprises me to the extent that it doesn’t surprise me anymore because I take it as a given’ (03).^9^

Several note how the incentive structure faced by policymakers creates an institutional short-termism at what we refer to as the meso-level. One, a senior epidemiologist with wide-ranging government advisory experience (07), urges that it is ‘very hard for Public Health England or US CDC to get sustainable funding year in year out’ because they focus on issues that concern the ‘population base’ rather than discrete events, and when emergencies occur, these are seen as ‘unpredictable events’ that are out of the ordinary. Against competing demands for funding, such bodies tend to lose out, but this short-termist leaning is the ‘political reality’. An intergovernmental civil servant (03) argues that World Bank and the WHO ‘are not incentivised to look at risk because the response of … international civil servants, is how do we respond to particular mandates given to us by member of states, how do we respond to the 24-h news cycle, and how do we respond to our nasty institutional competitors in other UN agencies who are going to get a bigger budget or a bigger mandate’. An international policy adviser (17) emphasises the extreme strength of the ‘political factor’ when ‘strategic interests are at stake’, such as where agreements of nuclear weapons are involved.

On the other hand, when pressed, interviewees would resist reducing their experience of policymaking to the outcome of large and raw expressions of competing power; that is, we might say, there is a resistance to reductivism to the macro- or meso-levels. One, who had held a government research role and had moved in to working in civil society (15), urges instead that limits to ‘bandwidth’ may be as much a factor in explaining the apparent short-termism of policy with regard to future catastrophic risk: ‘law makers are lot like single parents with three jobs. They don’t have a lot of scope for a long term thinking or anything other than the next thing.’ The realpolitik, then, is not necessarily taken as a hard limit on what may be achieved. Rather, it is another element of the complexity against which policy is to be made. Indeed, some emphasise the various actors at work and put their efforts in to identifying those who might be influenceable. We see this further below.

The attitude of awareness of complexity resulting from a multiplicity of actors has an occasional flip side: an accompanying scepticism about ‘top down approaches’ that begin by designing new ‘systems’ in the abstract. We might call this the Burkean mood of policymakers: ‘the reality about bureaucratic politics…it’s turf battles, it’s mission creep, it’s all these other things that go on. So no matter what lovely system you want to create, it doesn’t work’ (12). Another states, ‘we need to look at not a top-down approach, which somehow says what should and shouldn’t happen. We need to look at what will best incentivise a whole range of different actors doing things differently’ (03). Similarly, a leading policymaker in AI urges, ‘what we find is it’s quite difficult to have a conversation that’s very meaningful when talking in very general terms’ (22). We might make a rough generalisation in this area: those at the policy coalface, currently dealing with specific problems, are less likely to emphasise Realism about GCR policy and are more likely to adopt this Burkean mood, along with what we referred to above as the ‘Problem-solving mindset’, whereby focus is maintained upon what can conceivably be achieved in the near term by expanding on existing practices.^10^

### Theme three: influence

There is a strong narrative concerning the idea that individual policymakers can be a direct force for good in addressing GCR by doing their work well, including through the way that they deploy and manipulate policy concepts. The micro, individual level domain of power is emphasised. Sometimes this is expressed directly with reference to the GCR literature. One interviewee was influenced by theoretical work to bring the dangers of Artificial General Intelligence to the attention of decision-makers (16). Another (03) emphasises the importance of formative ‘micro-conversations’ in forming the conceptual schemes of those who will go on to hold power.

You’ve got…a lot of people a lot brighter than me who are… maybe going to end up in a sovereign wealth fund, maybe advising the government of Saudi Arabia or Abu Dhabi… If their framing of the world is just really, really slightly different and then they’re making a decision that relates to tens of billions of dollars being allocated in a particular area rather than on another, then perhaps that conversation is actually the most influential thing that I will have done. But I’ll never know.

As this suggests, one aspect of this narrative relates to the idea that language is where a great deal of the action is. This involves the idea that policymakers can make positive impacts by identifying ways of speaking and influencing the terms in which conversations about future risk are carried out. This point is illustrated in broader terms, as well as with specific reference to GCR. The broader point is well-expressed with an example put by one interviewee (12):

if…you work for the Ministry of Health and you go to see Treasury and you say “I need money for preparedness,” they’re going to say, “Well, what does that mean exactly? And what’s the line—what’s the return on that investment?” and lots of very pointed questions. And there’s no standard definition of preparedness from a financing budgeteer standpoint. That’s a reality, a practical reality and it’s born out of experience where you need to know your audience. So if your audience is the Ministry of Finance or your audience is some other sector, the problem is that these professional languages don’t communicate on the expectations and it’s as basic is that.

What comes across, then, is that there are imperfections and opportunities in the ways that professional languages function around future risk. Yet more strongly, the view is taken that early mastery of the language surrounding a new issue can lead to influence on the way that discussion of it will later run, through, as one interviewee puts it, the ‘creation of new thoughts’. In contrast, ‘you can rarely make a difference on issues where political positions have already crystallised, and it’s clear where the political lines are drawn’ (03). By skilfully defining the terms of a policy area, one can have an effect not just upon its efficacy but also its direction.

In the specific context of global catastrophic risk, there is a degree of ambivalence about the GCR ‘brand’, for these purposes. On one hand, one (16) takes the view that talk of GCR ‘scares’ senior leaders and would be seen as ‘too far out’ by those policymakers involved in risk mitigation, and that the term ‘tail end risk mitigation’ may closer map on to existing policy concepts. On the other hand, another (15), who has turned from civil service to non-governmental organisation work, states, “in…three to five years, it’ll be fantastic if I can approach a four star general or equivalent in the UK or in NATO, and say ‘I work on global catastrophic and existential risk’ and not be looked at like I’m a doomsday prepper.” In this regard, several interviewees pointed to the significant influence held speeches and articles by luminaries of the policy world (e.g., Kissinger, 2018).

Another aspect of the narrative of individual policymakers making a difference is the idea of what we might call the meso-level conservatism of the policy process, providing a resistance to phenomena arising from other levels. We noted above the way that there is a perceived short-termism in global governance of GCR that arises from the incentives that state actors and thereby policymakers face. Such short-termism may also be partly explained by habits or norms within the policymaking world. Thus, in discussing the International Health Regulations—a set of regulations that one might not expect to come about were states to act purely according to the narrative that they are self-interested in a short-sighted way—one interviewee (12), when asked why these regulations exist at all, referred to a ‘natural behaviour that people focus on the thing that’s in front of them’. That is, policy initiatives gain momentum, and once an area has resources and internal coherence, then it gains some resilience against forces that might diminish it. Similarly, another interviewee, working at the European Commission (13), offered an example of how a ‘foresight’ approach can encourage senior policymakers and politicians to take decisions in areas that they might otherwise cede to those with a technical focus. Such an approach is designed to reveal the values that are embedded in technicians’ current approaches; once these are more explicit, they fall under the instinctive remit of those with broader policy powers.

The strands of this narrative are brought together in a story told by an interviewee who works to influence legislators.^11^ A high-ranking member of staff of a member of Congress was persuaded by the argument that certain aspects of GCR ought to be taken more seriously. (‘The member…doesn’t really actually care or understand these issues’.) The interviewee describes how he was able to help the staffer to alter a small piece of language in a directive, such that a significant amount of money would be spent on ethics research relevant to this aspect of GCR.

The way that policymaking occurs, then, provides some optimism amongst policymakers that they can make a positive difference by working well, against a background in which changes in language can be highly significant, especially at the formative stages of a policymaking areas; in which policy ideas can form their own momentum; and in which ‘working well’ is construed as including following what is considered to be most important or valuable.

### Theme four: governance outside of Government

There is a narrative of positivity towards the possibility of creating institutions that govern GCR at the non-governmental level. As we have noted, governance is not just a matter of what governments do. This insight is often implicitly expressed through the idea that much effective governance of emerging technology will involve actions on the part of organisations that exist outside of state institutions. Our expert on emerging biotechnology policy (02) states, ‘where I find the most optimism is where I dream of creating supplementary spaces that create bridges between regulation and scientists that sit just under regulatory decision-making that augment the process.’ Another (12) refers to the importance of ‘regulations, codes of conduct, standards and norms’, noting that openness about data can encourage if not enforce compliance. Those funding academic or policy research, as well as advocacy, hold a degree of leverage in influencing future policy direction. Finally, emphasises another interviewee (03), there is the role of the global financial system: ‘probably, the locus of real decision-making has lied in … the private sector, the world financial institutions, development banks, individual companies, individual investment, and sovereign wealth funds and pension funds and insurance companies.’ While specific proposals and ideas are diverse, what comes across is that there are significant possible gains to be made at this international, non-governmental level, influencing both individual scientists at the micro-level and scientific meso-level governance.

Part of this narrative involves the idea that reform of the research world and its relation to policy would contribute positively to governance. One interviewee, who leads an NGO (08), imagines a possibility in which ‘we give incentives to academic scientists and scientists in the tech sector to come together with the policy community to come up with solutions to some of these problems, whether they’re technical solutions, whether they’re their behavioural solutions, whether they’re policy solutions’. Such people ‘have solved a lot more difficult things than this’. The problem is that the incentives of profit and tenure involve relatively little work on, for example, biosafety policy. And things could be otherwise: ‘we just have to create an environment for smart people to come up with those answers’. There are already in existence some small versions of such incentives, and these might be heavily intensified.

The way that good GCR governance straddles university and private sectors comes out forcefully in these conversations. Research on potentially transformative emerging technology is carried out both by those in academic and private sector roles, and indeed often researchers will work within each sector sequentially or both simultaneously. The incentives that technologists face again come to the fore in the following lines from a director of another NGO, with experience working with government:

you might have one lab, one university, or one country or region that has put certain safeguards in place to secure those technologies or to advance them and disseminate them in a way that reduces risk, but it’s not necessarily the case everywhere, and…if you individually do put regulations in place, in some cases, it might just encourage those businesses to go elsewhere where they don’t have those same regulations (14).

Ideas about reforms to the research world are not, however, universally put forward in terms of researcher incentives. A significant strand expresses the issue in terms of a cultural pattern of a misplaced deference on the part of policymakers to those with technical knowledge, and a misplaced implicit sense of expertise on policy matters held by those with a technical focus. One interviewee refers to the surprise of those at the centre of the ‘Gain of Function’ controversy that their research would be of public concern, stating, ‘a lot of scientists don’t want to be bothered’ (15). Another refers to the typical socio-economic background of those in science, and contrasts this with the undesirability of having policy that is ‘shaped by the lived experiences of a few’ (02). A further interviewee with experience of science policy both in government and in a large corporation expresses surprise at the similarities. Both are ‘bureaucratic’ and ‘siloed’, with a tendency of those with formal decision-making power towards conservatism and a dependence on technical assessments in the face of uncertainty (13).

## Discussion

We have analysed the outlook of those working in the governance of emerging technology that might pertain to a future catastrophic risk. Given its sample size, it is not an ambition of the study to provide, on its own, generalisable claims across all policymaking. The study’s themes nonetheless provide insights into the range and types of attitudes that are available. Although cynicism is far from unknown in the policy world, the degree of scepticism about existing structures for governing GCR is striking. This might provide encouragement on the part of those seeking reforms that they are on the right track. Furthermore, given that the scepticism applies across particular technology fields and policy problems, it provides reason for seeing the governance of GCR from emerging technology as a more unified problem, inhibited by common obstacles and susceptible to more general solutions.^12^ These obstacles occur at both the level of international governance, including difficulties in achieving the degree of international cooperation required for effective GCR governance, and at the level of domestic political drivers such as short-termism. The scepticism theme suggests that it would be fruitful to build upon studies of senior decision makers in climate change, where there has long been widespread acceptance among policymakers that climate change is a problem. The issue there isn’t how to persuade senior decision makers to accept the science; rather, it is how to understand why senior decision makers do not act decisively, even though they accept the science.

Let us return to the analytical frame that was introduced earlier, according to which, building on the Rickards et al. (2014) study of climate change governance, we can separate factors that bear on governance of global catastrophic risk into those occurring at the micro-level (individual and interpersonal factors), the meso-level (organisational and institutional factors), and the macro-level (societal, cultural, and economic factors). In the context of this framing, we can see the perceptions of policymakers highlighted in this study as shedding light on each of the three different levels, as summarised in Table 1 below.

**Table 1:** Governance obstacles and opportunities

	Obstacles	Opportunities
Micro	No new obstacles identified by participants at the micro-level	Individual action Facilitation of non-governmental organisations
Meso	Short-termist incentives upon individual policymakers Policy language Reform momentum Topic marginalisation	Policy language Reform momentum (Institutional reforms)
Macro	Economic competition Strategic competition	n/a

Consider the meso-level first. As we have seen, policymakers are aware of the powerful incentives that they and others face, and of the ways that such incentives can conspire to lead systems towards inefficient or short-termist policy. But such structures are not perceived as overwhelming the possibility of choice of action, and most indicate ways in which they individually can or have improved the workings of some aspect of governance. In particular, policymakers see opportunities to promote improvements by adopting the right forms of language and approaches to focus attention and financial resources on GCR governance issues. This is a response to a sense that a part of the reason that GCR governance is often overlooked is that it simply falls between the cracks, being deprioritised almost by accident rather than as a result of any underlying drivers, the result of overstretched political attention and time. In Table 1, then, the short-termist incentives upon individual policymakers at the meso-level are present, as are the opportunities for individual action. The effects of economic and strategic competition are present at the macro-level.

We have seen evidence that small, long-term risks of very bad outcomes may tend to be systemically marginalised because they do not tend to present themselves as immediate issues, they lack a conceptual scheme that is widely acknowledged amongst policymakers, and there is a conceptual conservativism in the policymaking process. Reform momentum, policy language and topic marginalisation all therefore arise as obstacles at the meso-level. Those obstacles may be addressed by those working within political institutions: individual policymakers can act directly as individuals at the micro-level against the tide of states’ strategic imperatives; languages and schemas may be developed at the meso-level that facilitate effective understanding on, for example, preparedness or global catastrophic risk; and policymaking inertia may be overcome and indeed reversed. The incentives that researchers face arose as a particular policy area within theme 4, ‘Governance outside of Government’, and we thereby report a perception of a governance opportunity here. Interestingly, countervailing new institutions, such as commissions for the future or other constitutional nudges towards long term thinking, did not naturally arise in discussions (institutional reforms accordingly placed in brackets in Table 1). The reform area of focus appears to be extra-governmental. It is deserving of further study how far (and if so, why) policymaker perceptions of effective reforms in this area avoid reforms of their own institutions.

It is helpful to understand the problem of achieving effective governance of GCRs as a two-pronged challenge. On the one hand, there are governance failures or inefficiencies that arise as a result of unintended policy marginalisation or momentum at a meso-level, but which could be remedied without confronting any entrenched obstacles arising from existing global or national power structures. Such difficulties are best addressed by developing new institutional mechanisms to ensure that GCR governance does not inadvertently drop off political agendas, and also by continuing efforts at solving particular problems of GCR governance, especially through global civil society efforts. In a similar vein, Boyd and Wilson (2020) recently urge a greater uptake at the level of global governance institutions, particularly throughout the UN, of the idea of confronting existential risks to humanity. On the other hand, there are problems of GCR governance that are inextricably linked to underlying issues in geopolitical and national governance, such as economic and military competition, and the short-termist pressures created by elections. Such problems appear more difficult to address, since they risk forcing confrontation with structures of power that have an interest in resisting changes. Opportunities at the macro-level are absent from our table because any change would be instigated by an individual or an institution. The challenge here implies that proposals for reform—such as representatives for future generations—must give a sufficiently robust account of how they propose to tackle the underlying issues of power that risk undermining them.

Our research also sheds light on the political changes necessary for effective governance might be achieved, by highlighting the opportunities possessed by those involved throughout the policy process. Here, the concern with the GCR brand, operating as a key issue at the meso-level, is worth highlighting. There is already some work that integrates insights from studies of GCR with existing policy concepts such as risk mitigation, risk assessment, and foresight studies (Avin et al., 2018; Government Office for Science, 2011); it would appear that there is a case for further work in this area insomuch as our findings suggests that such language is more likely to persuade political policymakers than the language of GCR. Promising further avenues of exploration would further compare how language and terminological choices have effects on the ways that policymakers approach matters of overwhelming disaster (Cohn, 1987; Sauer, 2015), and would also examine the ways that discussion of global catastrophe is best carried out at the nexus between policymakers and scientific experts.^13^ There is similarly emergent work on ‘national risk registers’, the values of which it is claimed include that they facilitate cooperation at the highest levels of government and that they provide objective measures of risks (OECD, 2018, but compare Hagmann & Cavelty, 2012). As we have seen, there is a wide range of actors and instruments that are relevant to GCR governance, straddling different forms of governance and also relating to different parts of the complex inter- and intra- disciplinary divides within the development of emerging technology. This complexity may undermine politically and sometimes render practically unhelpful the general schemas put forward by talk of GCR as a whole; however, if there is value in finding ways to assess and compare risks that appear to be of a qualitatively different kind across technology and policy areas, then there is a reason to develop GCR language.

This route to change is likely to be only a part of the jigsaw, however, since our research suggests that such influence can be effective for tackling GCR governance failures of the first type described above, but is less likely to provide an effective route for tackling failures of the second type, namely those linked to macro-level difficulties with global governance and political structures of power. In the latter case, more effective governance is likely to require the broader support of political actors or electorates, which in turn would require the development of a more general awareness of the threat posed by GCR and the governance failures that underlie such risks. It is worth noting here that, whereas the language of GCR may hinder efforts via the first route, it may be helpful for achieving the changes required by the second route, serving to raise public consciousness of the general category of GCR.^14^

Beyond the political domain, there is also scope to develop and improve governance mechanisms for GCR in other areas. A great deal of discussion surrounds ‘soft law’ and the culture of researchers and among policymakers (Marchant & Allenby, 2017). Within the biotechnology field, there is some momentum towards encouraging a research culture that is ‘non-hierarchical’ and draws extensively on public consultation (Rufo & Ficorilli, 2019). As we have noted, the current governance of AI is spearheaded, and even carried out, by individual industry initiatives for creating guidelines. Appraisals of these cultures by participants in the present study were not universally positive, and there may be opportunities to improve them.

## Conclusion

The research presented here demonstrates for the first time how interviews with those involved and close to policymaking around GCRs can shed critical new light on the nascent state of GCR governance. We found considerable scepticism about the suitability of existing governance mechanisms to address GCR among those interviewed, a degree of pessimism about underlying political obstacles, but also optimism that governance could be improved through various avenues for influence. Promising routes to improved governance appear to exist at governance areas outside of formal government, even in the absence of political leadership. Our intention here was to gather a broad overview of how those involved in GCR governance perceive the current state of play and options for improvement. The findings of the paper offer important new insights into how effective GCR governance mechanisms might be developed. In order to take forward this research agenda and to build a deeper understanding of routes to effective governance, we recommend more detailed research into how GCR governance might be implemented in different types of organisation, for particular technologies such as AI safety or DNA synthesis, and through specific policy processes. Future studies might also examine the other significant group who directly influence and will be impacted by governance of emerging technology, namely, technologists themselves.

## Footnotes

1 One commentator argues, ‘[global catastrophic risks and existential risks] arising out of nanotechnology, bioengineering, and AI are almost entirely unregulated at the international level’ (Wilson, 2013: 310).

2 Within the category of ‘global catastrophic risks’ we include existential risks, that is, threats that would eliminate the whole of humanity. To be sure, there are some strong arguments for the far greater badness of the end of humanity, in comparison to its being largely but not fully eliminated (Parfit 1984: pp. 453–4; Beard and Kaczmarek 2019). For the merely pragmatic reason that such a view is not widely internalised (Schubert et al., 2019), we broaden the focus to global catastrophes rather than restrict it to existential threats.

3 An exemplar of the anthropic bias is the following argument from a recent article in *Scientific American*: ‘Given the zero percent historical success rate of apocalyptic predictions, coupled with the incrementally gradual development of AI over the decades, we have plenty of time to build in fail-safe systems to prevent any such AI apocalypse’ (Sherner, 2017). Of course, if apocalyptic predictions had had historical success, then we would not be considering their probability.

4 Artificial General Intelligence’ is a subset of artificial intelligence. It is the programming of complex, human-like reasoning capabilities, such as problem-solving, planning, and learning, with the ability to work under uncertainty towards sophisticated goals (Bostrom 2014).

5 Rickards et al. themselves place some emphasis on the first two, noting that senior decision makers are somewhat homogenous, and concluding, “While the iron cage they have locked themselves in to is elevated, its bars occlude the view. Running hard at the centre of the treadmill of production, [senior decision makers] are too busy to notice the exhaust fumes spilling forth behind them. For this reason, climate change is a knowledge deficit problem…” (p. 766.).

6 We note that with its use of in-depth interviews, the study would fall within the definition of a ‘medium’ sized study in Braun and Clarke’s (2013) terms.

7 On the hope that such a study will be of use to policymakers, see Donmoyer (2012) — although we remain neutral on the ‘constructivism’ that Donmoyer supposes.

8 Where referred to in this text, each participant’s data ID code is placed in brackets.

9 On multistakeholderism, see Gleckman 2018

10 This generalisation is intuitive; nonetheless, its existence and contours might be established with further study. For example, how far does it persist (or indeed, does it tend to expand) with those who have left direct policymaking roles to take on broader advisory or NGO positions?

11 The data ID is suppressed here at the interviewee’s request.

12 This contrasts with, or even contradicts, the strand of thinking in the policy world that encourages focus on the details of a specific problem.

13 A coherent policy brand in this area may also promote an effective ‘whole of government’ approach (Christensen and Lægreid 2007). The present study is a part of a broader project that also examines the perceptions and attitudes of scientists and technologists regarding GCR.

14 Building on this thought, Baum (2015) identifies ways in which long term goals may be met with policies that also have short term or otherwise limited benefits.

## References

[CR1] Anderson S, Clarke V (2019). Disgust, shame and the psychosocial impact of skin picking: Evidence from an online support forum. Journal of Health Psychology.

[CR2] Avin S, Wintle BC, Weitzdörfer J, Ó hÉigeartaigh SS, Sutherland WJ, Rees MJ (2018). Classifying global catastrophic risks. Futures.

[CR3] Bauer A, Feichtinger J, Steurer R (2012). The governance of climate change adaptation in 10 OECD countries: Challenges and approaches. Journal of Environmental Policy & Planning.

[CR4] Baum SD (2017). On the promotion of safe and socially beneficial artificial intelligence. AI & Society.

[CR5] Baum SD (2015). The far future argument for confronting catastrophic threats to humanity: Practical significance and alternatives. Futures.

[CR6] Beard, S., and Torres, P., (2020). Identifying and assessing the drivers of global catastrophic risk: A review and proposal for the global challenges foundation. https://globalchallenges.org/assessing-the-drivers-of-global-catastrophic-risk-final/ (accessed May 2021).

[CR7] Beard S, Kaczmarek P (2019). On the Wrongness of human extinction. Argumenta.

[CR9] Beck U (1992). Risk society: Towards a new modernity.

[CR8] Beck U (2009). World at risk.

[CR10] Belfield, H. (2020). Activism by the AI community: Analysing recent achievements and future prospects. In Proceedings of the AAAI/ACM Conference on AI, Ethics, and Society (pp. 15–21). Chicago.

[CR11] Bostrom, N. (2014). Superintelligence: Paths, Dangers, Strategies. OUP Oxford.

[CR12] Bostrom, N. (2019). The Vulnerable World Hypothesis. Global Policy.

[CR13] Bostrom N (2011). Information hazards: A typology of potential harms from knowledge. Review of Contemporary Philosophy.

[CR14] Bostrom N (2013). Existential risk prevention as global priority. Global Policy..

[CR15] Boyd, M., & Wilson, N. (2020). Existential risks to humanity should concern international policymakers and more could be done in considering Them at the International Governance Level. Risk Analysis. July 10.1111/risa.13566 32691469

[CR16] Braun V, Clarke V (2006). Using thematic analysis in psychology. Qualitative Research in Psychology.

[CR17] Braun V, Clarke V (2019). Reflecting on reflexive thematic analysis. Qualitative Research in Sport, Exercise and Health.

[CR18] Caney S, González-Ricoy I, Gosseries A (2016). Political institutions for the future: A five-fold package. Institutions for future generations.

[CR19] Caplan B, Bostrom N, Cirkovic MM (2008). The totalitarian threat. Global catastrophic risks.

[CR20] Christensen T, Lægreid P (2007). The whole-of-government approach to public sector reform. Public Administration Review.

[CR21] Cohn C (1987). Sex and death in the rational world of defense intellectuals. Signs: Journal of Women in Culture and Society.

[CR22] Donmoyer R (2012). Can qualitative researchers answer policymakers’ What-works question?. Qualitative Inquiry.

[CR23] Esvelt KM (2018). Inoculating science against potential pandemics and information hazards. PLoS Pathogens.

[CR24] Farquhar S, Cotton-Barratt O, Snyder-Beattie A (2017). Pricing externalities to balance public risks and benefits of research. Health Security.

[CR25] Gartzke E, Kroenig M (2016). Nukes with numbers: Empirical research on the consequences of nuclear weapons for international conflict. Annual Review of Political Science.

[CR26] Geller, Daniel S. 2017. ‘Nuclear Weapons and International Conflict: Theories and Empirical Evidence’. In Oxford Research Encyclopedia of Politics.

[CR27] Gephart RP (2004). Normal risk: Technology, sense making, and environmental disasters. Organization & Environment.

[CR28] Gleckman H (2018). Multistakeholder governance and democracy: A global challenge.

[CR29] Government Office for Science (2011). Blackett review of high impact low probability risks. https://www.gov.uk/government/publications/high-impact-low-probability-risks-blackett-review. Accessed May 2021.

[CR30] Greaves, H., & MacAskill, W. (2019). The case for strong longtermism (No. 7–2019). Global Priorities Institute Working Paper Series. GPI Working Paper.

[CR31] Hagmann J, Cavelty MD (2012). National risk registers: Security scientism and the propagation of permanent insecurity. Security Dialogue.

[CR32] Hambira WL, Saarinen J (2015). Policy-makers’ perceptions of the tourism-climate change nexus: Policy needs and constraints in Botswana. Development Southern Africa.

[CR33] Jobin A, Ienca M, Vayena E (2019). The global landscape of AI ethics guidelines. Nature Machine Intelligence.

[CR34] Jones N, O’Brien M, Ryan T (2018). Representation of future generations in United Kingdom policy-making. Futures.

[CR35] Kemp, L. and Rhodes, C. (2020). The Cartography of Global Catastrophic Governance. https://globalchallenges.org/the-cartography-of-global-catastrophic-governance/ (accessed May 2021).

[CR36] Khan Z (2014). Pakistan’s nuclear policy.

[CR37] Kissinger, H. (2018). How the enlightenment ends.* The Atlantic*. https://www.theatlantic.com/magazine/archive/2018/06/henry-kissinger-ai-could-mean-the-end-of-human-history/559124/

[CR38] Klotz LC, Sylvester EJ (2014). The consequences of a lab escape of a potential pandemic pathogen. Frontiers in Public Health..

[CR39] Koffler N (2018). Editing nature: Local roots of global Governance. Science.

[CR40] Kooiman J (2003). Governing as governance.

[CR41] Kuhlemann K (2018). Complexity, creeping normalcy, and conceit: sexy and unsexy catastrophic risks. Foresight.

[CR42] Kurokawa T (2019). How to overcome the impasse on nuclear disarmament: An interview with Thomas Countryman. Journal for Peace and Nuclear Disarmament.

[CR43] Lentner HH (1976). Foreign policy decision making: The case of Canada and nuclear weapons. World Politics.

[CR44] Liu HY, Lauta KC, Maas MM (2018). Governing boring apocalypses: A new typology of existential vulnerabilities and exposures for existential risk research. Futures.

[CR45] Macnaghten P, Chilvers J (2014). The future of science governance: Publics, policies, practices. Environment and Planning c: Government and Policy.

[CR46] Manheim, D. (2020). The fragile world hypothesis: Complexity, fragility, and systemic existential risk. Futures.

[CR47] Marchant GE, Allenby B (2017). Soft law: New tools for governing emerging technologies. Bulletin of the Atomic Scientists.

[CR48] OECD. 2018. ‘National Risk Assessments: A cross Country Perspective. https://www.oecd.org/gov/national-risk-assessments-9789264287532-en.htm (accessed May 2021).

[CR49] Ord T (2020). The Precipice.

[CR50] Parfit D (1984). Reasons and persons.

[CR51] Perrow C (2011). Normal accidents: Living with high risk technologies.

[CR52] Pidgeon N, O’Leary M (2000). Man-made disasters: Why technology and organizations (Sometimes) fail. Safety Science.

[CR53] Pierre J, Peters BG (2020). Governance.

[CR54] Posner RA (2004). Catastrophe: Risk and response.

[CR55] Posner RA, Bostrom N, Cirkovic M (2008). Public policy towards catastrophe. Global catastrophic risks.

[CR56] Rickards L, Wiseman J, Kashima Y (2014). Barriers to effective climate change mitigation: The case of senior government and business decision makers. Wires Climate Change.

[CR57] Roberts A (2020). Bridging levels of public administration: How macro shapes meso and micro. Administration & Society.

[CR58] Robinson O (2014). Sampling in interview-based qualitative research: A theoretical and practical guide. Qualitative Research in Psychology.

[CR59] Rufo F, Ficorilli A (2019). From Asilomar to genome editing: Research ethics and models of decision. NanoEthics.

[CR60] Sagan SD (1996). Why do states build nuclear weapons? three models in search of a bomb. International Security.

[CR500] Sauer F (2015). Atomic anxiety: Deterrence, taboo and the non-use of U. S.

[CR61] Scherer MU (2015). Regulating artificial intelligence systems: Risks, challenges, competencies, and strategies. Harv. JL & Tech..

[CR62] Schubert S, Caviola L, Faber NS (2019). The psychology of existential risk: Moral judgments about human extinction. Scientific Reports.

[CR63] Shermer, M. (2017). Artificial Intelligence Is Not a Threat — Yet. March 1. https://www.scientificamerican.com/article/artificial-intelligence-is-not-a-threat-mdash-yet/

[CR64] Simonet G, Leseur A (2019). Barriers and drivers to adaptation to climate change—a field study of ten french local authorities. Climatic Change.

[CR65] Smallman M (2020). ‘Nothing to do with the science’: How an elite sociotechnical imaginary cements policy resistance to public perspectives on science and technology through the machinery of government. Social Studies of Science.

[CR66] Stedman RC (2004). Risk and climate change: perceptions of key policy actors in Canada. Risk Analysis.

[CR67] Taylor P, Bostrom N, Cirkovic MM (2008). Catastrophes and insurance. Global catastrophic risks.

[CR68] Tonn B (2009). Beliefs about human extinction. Futures.

[CR69] Tonn BE (2018). Philosophical, institutional, and decision making frameworks for meeting obligations to future generations. Futures.

[CR70] Turner J (2018). Robot rules: Regulating artificial intelligence.

[CR71] Wiener, J. B. (2016) The tragedy of the uncommons: On the politics of apocalypse. Global Policy 7.S1 (2016), 67–80

[CR72] Wilson G (2013). Minimizing global catastrophic and existential risks from emerging technologies through international law. Virginia Environmental Law Journal.

[CR73] Wynne B (1993). Public uptake of science: a case for institutional reflexivity. Public Understanding of Science.

[CR74] Wynne B (2001). Creating public alienation: expert cultures of risk and ethics on GMOs. Science as Culture.

[CR75] Yoshida F (2018). From the reality of a nuclear umbrella to a world without nuclear weapons: an interview with Katsuya Okada. Journal for Peace and Nuclear Disarmament.

[CR76] Yudkowsky E, Bostrom N, Cirkovic MM (2008). Cognitive biases potentially affecting judgment of global risks. Global catastrophic risks.

